# Southeastern European Health Network (SEEHN) Communicable Diseases Surveillance: A Decade of Bridging Trust and Collaboration

**DOI:** 10.3402/ehtj.v6i0.19950

**Published:** 2013-01-25

**Authors:** Silvia Bino, Semra Cavaljuga, Angel Kunchev, Dragan Lausevic, Bernard Kaic, Adriana Pistol, Predrag Kon, Zarko Karadjovski, Stela Georghita, Snezana Cicevalieva

**Affiliations:** 1 Regional Development Center of Communicable Diseases Surveillance and Control, Institute of Public Health, Tirana, Albania; 2 Faculty of Medicine, University of Sarajevo, Bosnia and Hercegovina; 3 Ministry of Health, Sofia, Bulgaria; 4 National Institute of Public Health, Podgorica, Montenegro; 5 National Institute of Public Health, Zagreb, Croatia; 6 Institute of Public Health, Bucharest, Romania; 7 Institute of Public Health, Belgrade, Serbia; 8 Institute of Health Protection, Skopje, Macedonia; 9 Ministry of Health, Chişinǎu, Moldova; 10 Ministry of Health, Skopje, Macedonia

**Keywords:** SEE, SEEHN, communicable diseases surveillance network, South-eastern Europe

## Abstract

The communicable disease threats and changes that began emerging in south-east Europe in the early 1990s – after a decade of war and while political and health systems region-wide were undergoing dramatic changes – demanded a novel approach to infectious disease surveillance. Specifically, they called for an approach that was focused on cross-border collaboration and aligned with European Union standards and requirements. Thus, the Southeastern European Health network (SEEHN) was established in 2001 as a cooperative effort among the governments of Albania, Bosnia and Herzegovina, Bulgaria, Croatia, Moldova, Montenegro, Romania, Serbia, and the former Yugoslav Republic of Macedonia. In 2002, SEEHN initiated a communicable diseases project aimed at strengthening both national and regional surveillance systems with a focus on cross-border collaboration. Over time, SEEHN has nurtured growth of a regional fabric of SEE experts in communicable diseases surveillance and response who are able to discuss emerging issues and best practices at any time and without being constrained by the rigidity of traditional or existing systems. Main achievements to date include joint preparation of influenza pandemic preparedness plans at both national and regional levels and the introduction of molecular techniques into influenza surveillance laboratories region-wide. Here, we describe the history of the SEEHN communicable disease project; major activities and accomplishments; and future sustainability of the regional infectious disease surveillance network that has emerged and grown over the past decade.

## Introduction

Communicable diseases continue to pose a threat for southeastern European countries. New infectious diseases are emerging, old diseases are reappearing (e.g., tuberculosis, West Nile Fever etc.), and incidences for many diseases are rising (e.g., HIV and other sexually transmitted infections) (1–5). Much of this evolving disease burden began in the early 1990s, when transition toward a market economy led to widespread social unrest and when civil wars in the region displaced large numbers of people and created populations that were vulnerable to communicable diseases and difficult to reach through existing health care systems (6–9). Health care reforms and the privatization of previous public services led to the technical and political isolation of many national public health institutes and services and to a lack of coordination of cross-border activities. Systemic reporting of infectious disease events was lacking in some countries; outbreak investigations were inefficient; and surveillance practices were poorly financed, outdated, and without the flexibility needed to respond to the wide range of health threats emerging during that time. Added to these regional challenges, new global markets have facilitated the spread of infectious disease (10).

These regional and global changes demanded a novel approach to infectious disease surveillance at the regional level – one well integrated with national systems, focused on cross-border collaboration, and aligned with European Union standards and requirements. To achieve that end, in 2002 the Southeastern European Health Network (SEEHN) initiated a communicable diseases project aimed at strengthening regional surveillance via a network of experts, communicable diseases surveillance officers, ministries, public health institutes, and universities. This paper describes the history and governance of SEEHN, with a focus on its communicable disease project (one of three major SEEHN projects); major activities and accomplishments of the SEEHN communicable diseases project; and strategies for sustainability.

## History and Governance

The Southeastern European Health network (SEEHN) is a cooperative effort among the governments of Albania, Bosnia and Herzegovina, Bulgaria, Croatia, Moldova, Montenegro, Romania, Serbia, and the former Yugoslav Republic of Macedonia. Established in 2001 as the health component of the Stability Pact for southeastern Europe, SEEHN operates at political and technical levels to enhance regional cooperation and coordination in public health and to promote sustainable development of SEEHN member states by improving people's health (11).

### The Stability Pact

The Stability Pact itself was established in 1999, in response to a decade of conflicts, war, humanitarian emergencies, and economic crises throughout the region. The goal of the Stability Pact was to promote stability and reconciliation in the region through three major sets of activities: (i) democratization and human rights, (ii) economic reconstruction, cooperation and development, and (iii) security (12). The role of the Stability Pact has changed over time. Originally, it served mostly as a platform to channel funds and coordinate donors’ activities. Over time, it evolved into a forum for member countries and international partners to convene on an equal basis in order to identify common problems and devise shared strategies for addressing those problems.

Public health was viewed as an uncontroversial area that could have an especially significant impact on strengthening regional social cohesion. Thus, in 2000 the ministries of health of Albania, Bosnia and Herzegovina, Bulgaria, Croatia, Macedonia, Romania, and the former Serbia and Montenegro convened a preliminary meeting in Sofia to discuss the wisdom of collaborating in regional and cross-border public health and adding health into the social cohesion agenda of the Stability Pact. In 2001, the ministries of health of all Stability Pact member countries requested that the World Health Organization (WHO) Regional Office for Europe, in collaboration with the Council of Europe, organize the First Forum of Health Ministers of South Eastern Europe. That meeting, held in Dubrovnik, Croatia, resulted in signing of *The Dubrovnik Pledge* (13), which laid out several specific objectives: increase citizens’ access to appropriate, affordable and high-quality health care services; intensify social cohesion by strengthening community mental health services; increase the quality of and regional self-sufficiency in the provision of safe blood and blood products; develop integrated emergency health care services that are offered free of charge to the user; strengthen the surveillance and control of communicable diseases; strengthen institutional capacity and intersectoral collaboration for access to affordable and safe food products; and establish regional networks and systems for the collection and exchange of social and health information. The pledge ended with a call to international donors for financial assistance and to the WHO Regional Office for Europe and the Council of Europe for technical and policy support. The SEE Health Network (SEEHN) was set up later that year to ensure implementation of *The Dubrovnik Pledge*.

Communicable diseases was one of public health topics chosen by SEEHN as an area of concentration. A regional communicable diseases project office was established in Tirana, Albania, near the Institute of Public Health; and a regional network of experts, communicable diseases surveillance officers, ministries, public health institutes, and universities was formed. Over time, the project has also benefited from input from partners from other regions of Europe, such as the Institut de Veille Sanitaire (INVS) in France, the National Public Health Institute of Slovenia, and the Center of Diseases Control in Greece or other institutions in Belgium and UK.

The first SEEHN communicable diseases project meeting was held in Vlora, Albania, in August 2002. Representatives from each member country met to review existing surveillance and early warning systems for the timely detection and control of epidemics and unusual events, ways to strengthen national and cross-border surveillance, and ways that national surveillance systems could be better linked to regional and global alert and response networks. Meeting attendees established principles of cooperation for the newly formed communicable diseases surveillance network (see below); appointed leading coordinators for the network from each country in consultation with respective ministries of health (with the expectation that leading coordinators would be supported by teams of experts in various animal and human health areas); and proposed a strategic plan for the network (see below) (14).

Since that first meeting in Albania in 2002, a commitment to long-term collaboration, coupled with the emerging risk of pandemic influenza and the mandate of the revised IHR, have driven new efforts to strengthen cross-border communicable diseases control across southeastern Europe. Most recently, a Regional Health Development Center (RHDC) on Communicable Disease was established at the Institute of Public Health, Tirana, Albania, in November, 2010.

### SEE Communicable Diseases Surveillance Network: Principles of Cooperation

Attendees of the 2002 SEEHN communicable diseases project meeting established the following principles of cooperation for the newly formed surveillance network: ownership by countries of Southeastern Europe (SEE); partnership approach; equal involvement of SEE countries; equal distribution of activities and resources; sustainability (SEE ministries of health commitment to project implementation at national level, capacity-building, and mobilizing of resources for further expansion); complementary and continuity (which implies building up ongoing plans); establishment of fixed funds allocated to management; decentralization of resources; transparency and accountability; overall management by a coordinator and a multi country steering committee; and regular reporting by the coordinator and steering committee to the network.

### The SEE Communicable Diseases Surveillance Network Strategic Plan

The four phases of the SEEHN communicable diseases project strategic plan are: (i) strengthen national surveillance systems through prioritization, evaluation, coordination and integration mechanisms and through training in applied field epidemiology; (ii) establish national policies and guidelines for communicable disease surveillance systems and response protocols for outbreaks that are tailored to country and subregion needs but also compatible with European Union approaches and procedures; (iii) strengthen integrated laboratory surveillance capacities and information exchange mechanisms, with special emphasis on influenza and national influenza preparedness plans; and (iv) develop and deepen regional cooperation, with a focus on common cross-border technical capacity required to deal with potential outbreaks and ensuring proper implementation of the 2005 IHR.

## Major Activities

Over the past decade, the SEE communicable diseases surveillance network has undertaken several major activities in accordance with the four phases of the strategic plan, depending on country and subregion needs.

### Phase 1: Human Capacity to Conduct Surveillance

The first activities of the SEEHN communicable diseases network were aimed at strengthening regional institutional and human capacities in public health surveillance and safety and health protection standards region-wide. Many of these activities were trainings. SEE surveillance officers and other public health specialists participated in an introductory course conducted by the European Programme for Intervention Epidemiology Training (EPIET) and an international applied epidemiology course conducted by Emory School of Public Health (Atlanta, Georgia, United States) and U.S. Centers for Disease Control (CDC). Additionally, regional experts collaborated with EPIET, WHO, and INVS to prepare a package of introductory training materials in applied epidemiology and surveillance system management. The package was distributed, translated as necessary, and used to train a number of communicable diseases surveillance managers across the region. Other trainings were conducted as necessary on data management and information systems, geographic information systems (GIS) and public health mapping, second generation HIV surveillance, and other topics of national or cross-border relevance.

Additionally, a prioritization exercise to determine the most important issues related to surveillance and response of communicable diseases was organized in November 2002 in Bucharest, Romania. Afterward, many of the countries performed a rapid evaluation of their own national needs in field epidemiology and ways to use existing trainings in their own curricula and practice. Based on the rapid evaluations, some countries prepared their own national introductory and applied field epidemiology courses in their national languages using national or subregional case studies (15).

These various early training activities were followed in 2003 and 2004 with assessments and analyses of communicable surveillance systems and early warning systems to streamline efforts on how to improve and integrate national surveillance systems in Albanian, Bulgaria, Moldova, Macedonia, and the former Serbia and Montenegro. In collaboration with WHO and other European experts, SEE experts prepared and implemented the assessments at national institutions and at SEEHN regional meetings in Romania and Bulgaria.

This early period of network activity was characterized by increasing contact among specialists and growing coordination among SEE countries; exchange of information on epidemiological situations and other irregular events; joint planning of future surveillance systems reforms; networking among public health and other relevant institutions, including laboratories; and the promotion of regional expertise in communicable disease surveillance and response. Since then, the network has held annual meetings, usually in country member capitals, with technical support provided by WHO experts and other consultants.

### Phase 2: National Policies and Guidelines

Major activities undertaken during the second phase of the SEEHN communicable diseases project included: harmonization of guidelines, case definitions, and procedures with EU country standards; preparation of national policies and guidelines on communicable diseases surveillance systems and outbreak responses; revision and adaptation of new legislation on communicable disease issues; assessment of national influenza surveillance systems; preparation of national influenza pandemic preparedness plans; translation of the revised IHR into member country languages; and development of initial work plans on IHR implementation. While not all member countries completed all tasks, almost all member countries prepared national guidelines on communicable diseases surveillance and response and adapted their case definitions to align with EU standards. Launching of the revised IHR prompted different actions in different countries, with some countries preparing fact sheets and other tools to help with implementation.

### Phase 3: Laboratory Capacity and Information Exchange

The third phase of the strategic plan addressed actual emerging problems, especially avian influenza. The network sought to increase regional capacity to rapidly detect clusters of human cases of avian influenza (Text Box 1) and monitor the spread of avian influenza viruses in both human and animal populations by improving integrated surveillance systems and building laboratory capacity (Text Box 2). Specifically, the network collaborated with WHO and the newly established European Centre for Disease Prevention and Control (ECDC) to manage a regional network of influenza experts. SEE influenza experts also participated in European influenza meetings in other regions and began sharing knowledge, experience, and information with experts from other areas of Europe. Additionally, SEEHN organized and coordinated joint trainings in influenza laboratory methodologies, with a focus on rapid diagnostic molecular techniques; and developed laboratory methodology training materials for use among experts with varying levels of experience in molecular techniques. Finally, a regional influenza diagnostic center was opened at the Cantacuzino Institute, Romania.

***Text Box 1.* A SEE regional assessment of national pandemic preparedness**

A major activity of the third phase of the SEEHN communicable diseases surveillance network was an assessment of pandemic preparedness among all member countries. Country assessment visits were organized and agendas set by national contact persons. Each country was visited by a team of experts from various agencies, including WHO, ECDC, and the UK Department of Health. Comprised of experts from different areas of pandemic preparedness, the teams used an assessment methodology that had been previously applied in EU Member States (in 2006–7). The assessments involved meetings with various national and local government agencies and with regional institutes of public health to gather information on gaps, challenges, and opportunities for further improvement in pandemic preparedness. Topics addressed included planning and coordination; communication; situation monitoring and assessment; health system preparedness, including surveillance and laboratory readiness; pharmaceutical and non-pharmaceutical interventions; general society preparedness; local level preparedness planning; interoperability; and pandemic exercises. Observations made during the country assessment visits were presented in a workshop on pandemic preparedness in Romania in 2008.

After the assessment it was concluded that while a great deal of work in pandemic preparedness had been completed and much progress had been made, some components had not been addressed yet and others needed to be revised and strengthened. SEE countries and their partners were focusing efforts on strengthening avian influenza contingency plans; while doing so is a crucial step to containing the spread of a new human influenza virus, it is not the only step to pandemic preparedness. Recommendations were made for preparedness in other realms outside of the health sector; for regional collaboration; and for additional preparedness exercises.

***Text Box 2.* Building SEE molecular diagnostic laboratory capacity**

Another significant component of the third phase of the SEEHN communicable diseases surveillance network strategic plan was building molecular diagnostic laboratory capacity. During the 2009–2010 H1N1 inflienza pandemic, hundreds of thousands of samples were tested in SEE laboratories in Tirana, Albania; Zagreb, Croatia; Belgrade and Novi Sad, Serbia; Romania; Bulgaria, Skopje, Macedonia; and Sarajevo and Banja Luka, Bosnia and Herzegovina. Virologists and laboratory technicians responsible for the testing had been trained during the previous years using capacities developed by the SEEHN communicable diseases surveillance network. During the course of the pandemic, they shared their experiences with each other weekly and at meetings. Additionally, the Subregional Influenza Laboratory Centre at the Cantacusino Institute in Romania played a crucial role in helping several countries to document the beginning of the pandemic and in communicating to countries the importance of sharing subregional capacities.

## Phase 4: Regional Cooperation

Much of the most recent network activity has revolved around strengthening regional cooperation. Many activities (especially during the 2008–2010 period) continue to focus on avian and other pandemic preparedness. These include: preparation of packages for assessing pandemic preparedness and response in each country, in collaboration with WHO and ECDC; assessment of pandemic preparedness in all SEE countries, again in collaboration with WHO, ECDC, and other international experts (Text Box 1); evaluations of the scale of the H1N1 pandemic in South East Europe and the burden of that pandemic on health systems in SEE countries; and evaluation of the use of pandemic preparedness plans in implementing the revised IHR. Plus, all SEE countries now participate in EuroFlu, a regional influenza surveillance platform launched by the WHO European region office in 2008.

In addition to its focus on pandemic preparedness, in 2008 a meeting was held in Zagreb, Croatia, to address the impact of the SEEHN communicable diseases surveillance network on IHR implementation. Following the meeting, national and subregional plans were developed to strengthen laboratory capacity and early warning systems for emerging and reemerging diseases. The plans were then tested in table top exercises in collaboration with the Health Protection Agency. At another meeting, experts from Macedonia, Albania, Italy, Greece, and the United Kingdom developed a SEE regional action plan for brucellosis surveillance and response (16, 17). In 2011, yet another meeting was held in Tirana, Albania, to share experiences and discuss potential solutions to a reemergence of measles in some SEE countries and lack of access to vaccination among some subpopulations (18–20). The meeting led to discussion of establishment of a web-based platform for sharing immunization data.

## Key Achievements

The main achievement of the SEEHN communicable diseases project has been establishment of a regional fabric of experts in different fields of communicable diseases who are able to discuss emerging issues and best practices at any time and without being constrained by the rigidity of traditional or existing social structures. Over a decade of collaboration, network members have trained together, shared a wealth of collective experience, resolved difficult challenges, and learned the value of working together in pursuit of a common goal. The case studies presented in Text Boxes 1 and 2 illustrate how pooling resources across countries benefits not just the SEE region, but also bordering countries. Additionally, network members have served as expert consultants for various WHO, ECDC, and other infectious disease surveillance activities conducted in the SEE region, such as the pandemic preparedness assessments described in Text Box 1.

In addition to introductions of pandemic preparedness across the region (Text Box 1) and molecular techniques into influenza surveillance laboratories region-wide (Text Box 2), other major specific accomplishments include: training of more knowledgeable and better prepared communicable diseases officers (e.g., through applied epidemiology trainings and cross-border field outbreak investigations); improvement of national surveillance systems (e.g., establishment of national communicable diseases surveillance centers or strengthening of existing centers, introduction of surveillance problems into health care reforms); use of pandemic preparedness to strengthen general emergency preparedness; increased collaboration between animal and human sectors; and use of the network to improve initiation of IHR implementation in SEE countries.

## Moving Forward

In 2008, the Stability Pact was transformed into its successor organization, the Regional Cooperation Council. This was in response to the need for a more regionally owned framework to reflect the substantial progress on the ground that had been achieved since the Stability Pact's origin and improving political, economic and social conditions across SEE. With full commitment and support from SEE countries, donor countries and other international actors, the Regional Cooperation Council inherited the mandate of the Stability Pact. As part of the transition to regional ownership, new terms for SEEHN operation, its structure, responsibilities, and funding mechanisms were laid out in the *Memorandum of Understanding on the Future of the South-eastern Europe Health Network in the Framework of the South East European Co-operation Process (2008 and beyond)* (21).

Today, SEEHN communicable diseases surveillance network members are fully committed to continuing and strengthening the collaboration that has been building over time. In addition to all member countries being fully committed to the new terms laid out in the *Memorandum of Understanding*, the *Memorandum* outlined steps for establishing Regional Health Development Centers (RHDCs). One of these centers was the previously mentioned RHDC on communicable diseases surveillance and control in Tirana, Albania. The center in Tirana is not only a legacy of the SEEHN communicable diseases surveillance network efforts over the past ten years, but it also represents transformation of past work into a long-term program of regional cooperation on communicable diseases surveillance and control and IHR implementation.

Additionally, the network derives strength from participation in Connecting Organizations for Regional Disease Surveillance (CORDS). Like other CORDS members, the SEEHN communicable diseases network is built on principles of trust and collaboration. CORDS interactions help to nurture both behaviors. Also through CORDS, SEEHN communicable diseases surveillance network experts regularly exchange information and share best practices with like-minded infectious disease networks, like the Middle East Consortium of Infectious Disease Surveillance (MECIDS), operating in other areas of the world.

Moving forward, partially drawing on the strength derived from CORDS, the SEEHN communicable diseases network will continue to address gaps in SEE regional infectious disease surveillance. For example, while all SEE countries participate in EuroFlu, not all SEE countries consistently report data. Plus, only four SEEHN countries conduct routine surveillance of severe disease due to influenza (Severe Acute Respiratory Infection, or SARI): Albania, Moldova, Romania, and Serbia. These gaps in influenza surveillance need attention.
